# Polymer Nanocomposites—A Comparison between Carbon Nanotubes, Graphene, and Clay as Nanofillers

**DOI:** 10.3390/ma9040262

**Published:** 2016-04-01

**Authors:** Mrinal Bhattacharya

**Affiliations:** Department of Bioproducts and Biosystems Engineering, University of Minnesota, St. Paul, MN 55108, USA; bhatt002@umn.edu; Tel.: +1-612-625-5234

**Keywords:** polymer nanocomposites, carbon nanotubes, graphene, clay, properties

## Abstract

Nanofilled polymeric matrices have demonstrated remarkable mechanical, electrical, and thermal properties. In this article we review the processing of carbon nanotube, graphene, and clay montmorillonite platelet as potential nanofillers to form nanocomposites. The various functionalization techniques of modifying the nanofillers to enable interaction with polymers are summarized. The importance of filler dispersion in the polymeric matrix is highlighted. Finally, the challenges and future outlook for nanofilled polymeric composites are presented.

## 1. Introduction

The use of fillers for the enhancement of polymer properties has been well documented. Initially, fillers were used to reduce the cost of the polymeric products. However, with time, fillers became an integral part in many applications, particularly for reinforcing the mechanical properties of the polymer. “Reinforced” polymers consist of a polymeric matrix and a relatively stiff inorganic filler that undergoes dramatic change in modulus or stress at given strain over the pure polymer. Traditional fillers include talc, glass fibers, carbon black, and calcium carbonate particles in the micrometer range. However, most micron sized traditional fillers require high loading for modest property enhancement, causing problems in melt flow and processing due to the high viscosity of the filled materials. Furthermore, the high density of traditional fillers also leads to heavier composites. Finally, the lack of interfacial interaction between the filler and the polymeric matrix leads to weak interfacial adhesion and results in failure.

A broad diversity of filler sizes has been used in reinforcing polymeric matrix. Edwards [1] in his review for filler reinforcement observed that “there is, nevertheless, good evidence that small particle size is a necessary requirement, and very likely the predominant requirement, for the reinforcement effect in rubber”. High degree of reinforcement is observed in the particle size range of 100 nm and below [1]. It has been reported that nylon 6 required three times more mass of glass fibers than clay montmorillonite (MMT) platelets to cause a doubling in the modulus [2]. Nanofillers in the range of 3%–5% by weight achieve the same reinforcement as 20%–30% of microsized fillers. Thus, nanocomposites have a weight advantage over conventional composites and, nanoscale materials have emerged as an attractive candidate as fillers as their increased specific interfacial area enables potentially higher interfacial interactions and hence, higher modulus.

**Figure 1:**
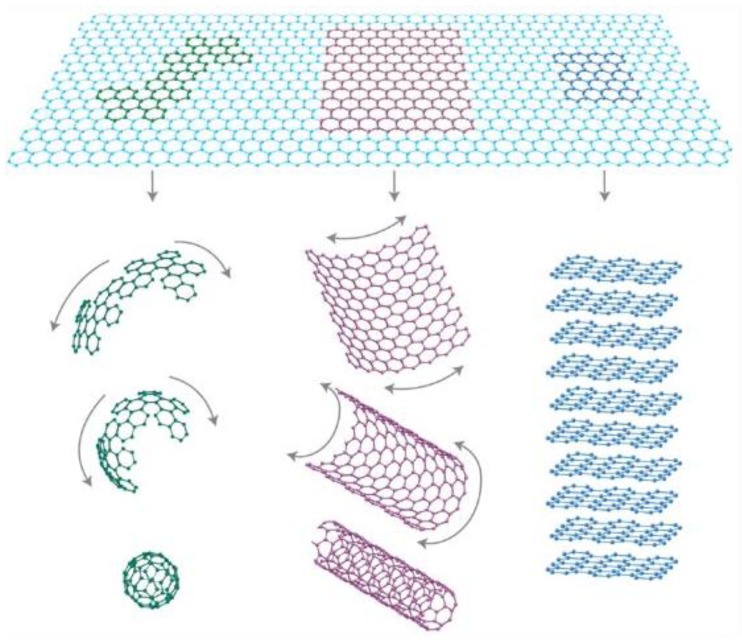
Graphene is a 2D building material for carbon materials of such as 0D buckyballs, 1D nanotubes or 3D graphite. Reproduced from Reference [13] with permission.

Nanofillers can be categorized on the basis of their dimensions—one dimensional which include nanotubes and nanowires [3,4], two dimensional such as nanoclays [5] and graphene [6], and three dimensional such as spherical [7] and cubical nanoparticles [8]. Carbonaceous nanofillers such as nanotubes and graphene display excellent properties due to their high mechanical strength and high aspect ratio. Graphene is a two-dimensional single atom thick sheet composed of sp^2^ carbon structure arranged in a honeycomb structure (Figure 1). It can be considered as a fundamental building block for all sp^2^ hybridized carbon allotropes. It can be wrapped up into 0D fullerenes, rolled into 1D nanotubes or 3D graphite when a number of graphene layers stack up. The 2D geometry of graphene nanosheets is responsible for the maximum value of its surface to volume ratio. These are favorable for good reinforcement of polymers.

Carbon nano tubes have attracted attention because of their unusual structures and properties. High Young’s modulus in the direction of the nanotube’s axis, electrical conductivities that vary from insulating to metallic, and their hollow structures are attractive features. Carbon nanotubes are half as dense as aluminum and have tensile strengths 20 times that of high strength alloys [9]. Studies [10,11] have shown that nanotubes display extraordinary mechanical properties—tensile modulus of 1 TPa, tensile strength in the range of 50–150 GPa and a failure strain in excess of 5%.

A graphene nanosheet with a Young’s modulus of 1 TPa and ultimate strength of 130 GPa is one of the strongest materials known [12]. It has a high specific area of 2600 m^2^/g, very high electric conductivity (6000 S/cm) [13], thermal conductivity (~5000 W/mK) [14] and high gas impermeability [15].

**Figure 2:**
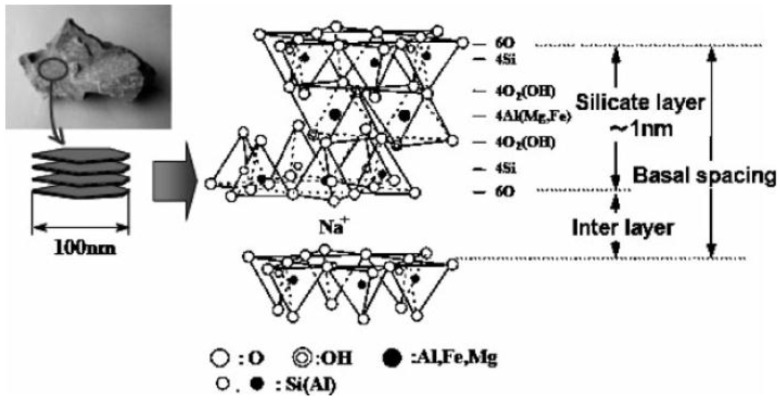
Structure of montmorillonite. Reproduced from reference [5] with permission.

Clays are naturally found as platelets, stacked from a few to as many as one thousand sheets. A single sheet of MMT was reported to have an in plane Young’s modulus ranging between 178 and 265 GPa [16,17]. These excellent properties of nanofillers make them suitable candidates for reinforcing polymer matrix. Montmorillonite (MMT), is the most widely used clay nanofiller, sandwiched between two silicate layers of an octahedral sheet of alumina. The nanometer-scale sheets of aluminosilicates have dimensions of 1–5 nm thickness and 100–500 nm in diameter (Figure 2). These dimensions lead to platelets of high (>50) aspect ratio. Hence, when blended with polymer, it enables stress transfer from the polymer to the mineral. The stiffness of the clay minerals results in increased mechanical properties of the blend.

This article reviews the processing techniques for developing nanocomposites and summarizes their properties. The focus will be between 1D *versus* 2D carbonaceous nanofillers (nanotubes *versus* graphene), and between two different 2D nanofillers (graphene *versus* clay). We limit the discussion to 1D and 2D fillers as it has been reported that 2D fillers provide higher degree of reinforcement than spherical shaped fillers [18].

## 2. Considerations for Developing Nanocomposites

Because of the contrast in composition, interaction, and properties between dissimilar components in nanocomposites, several key factors affect the role that nanoparticles play as reinforcing fillers in a polymer-matrix. For example: (i) fillers should have excellent mechanical properties such as strength and Young’s modulus; (ii) they should have high aspect ratio and high surface area to enable interaction with the polymer; and (iii) they should be well dispersed and avoid agglomeration.

The dispersion of nanoparticle in the matrix to the point where individual particles are coated by the polymer is extremely critical. Increased dispersion help achieve good load transfer to the nanofiller network, resulting in more uniform stress distribution. The mismatch between the properties of nanoparticle and polymer is mitigated by the increased interfacial area between the filler and matrix, leading to improved strength. Alignment of the filler in the matrix, while important, is not critical. While alignment maximizes modulus and strength, it makes the composite anisotropic. Bonding between the filler and the polymer is essential to allow the external stress applied to the composite to be transferred to the nanofillers, enabling them to bear most of the applied load. Hence, nanoparticles are often functionalized to improve their dispersibility, and enable their interactions with polymers. In addition to criterion listed above, the cost of nanomaterial is also an issue.

Several mathematical models such as, Kerner, Nielsen, Halpin-Tsai, and Mori-Tanaka [19,20], developed for conventional composites, can be used to obtain a qualitative measure of Young’s modulus of the nanocomposites based on filler geometry, dispersion, and interfacial properties. Results from these models indicate that as the filler content increases, the Young’s modulus increases linearly though the differences in the predicted values among various models can easily reach 200%. Spherical particles have a much weaker reinforcing effect on the nanocomposites properties. Randomly distributed platelets result in the strongest isotropic nanocomposites, while aligned fibers or platelet exhibit direction dependent reinforcement [21]. Dramatic changes in mechanical properties are reported to be obtained at low loadings (~2% or less) using exfoliated nanoclays [22,23], graphite nanoplatelets [24], and carbon nanotubes [25].

In addition to strong mechanical performance, nanocomposites also provide improved optical, thermal, and barrier properties. For example, clay nanoparticles possess excellent optical transparency [26,27], barrier properties, and fire retardency [28,29]. Carbon and metal nanoparticles in addition to reinforcing the polymer, also add electrical conductivity, catalytic activity, and plasmonic properties [30].

## 3. Interfacial Interactions between Filler and Polymer

Mechanical performance of polymer nanocomposites is dependent upon the interfacial interaction between the nanofiller and the polymer matrix. TEM micrographs on fractured surfaces showed nanotube pull-out and nanotube fracture as well as bridging by nanotubes [31]. This implies that to utilize the reinforcing capability of carbon nanotube (CNT) and maximize the mechanical properties of the composites, strong interfacial bonding is necessary. If the interfacial region is stronger than the matrix, the matrix will yield. However, if the interfacial region is weaker than the matrix, de-bonding may initiate along the interface. The extent of interaction would depend on how well the filler is dispersed in the matrix. Because of the strong van der Waals forces and electrostatic interactions, nanofillers tend to aggregate in solvents. Although van der Waals forces are considered to be weak intermolecular forces, they become significant at the nanoscale due to the large surface area per unit mass of the material. Nanotubes tend to self-assemble along the length axis. Agglomerated nanotubes form ropes that slip when stressed due to their poor adhesion to the polymer matrix, affecting their elastic properties [3,32,33]. Nanotube bundles act as stress concentration points within the polymer matrix and can in some cases, reduce the mechanical properties of the original polymer. The reduced aspect ratio also leads to a reduction in reinforcement. Graphite is composed of many graphene sheets held together by van der Waals forces to form rigid platelets several hundreds of nanometers thick. Since most of the graphene sheets in the stacks are unable to interact with the polymeric matrix, its performance is limited. Similar issues arise in the case of clay. In its pristine state, clay exists as stacks of many platelets. In aqueous solution, the hydration of the sodium ions causes the clay to swell and disperse the platelets which allows it to achieve increased interfacial or surface area to interact with polymer. The interaction between the clay and polymer occurs through ion-dipole bonding to the exchangeable cation on the surface of the clay.

Extensive work has been conducted in dispersing nanotubes in solvents. The dispersion procedure involves sonication in a bath for a period of time. The mechanical energy generated during sonication overcomes the van der Waals forces between the nanotube bundles or graphene/clay platelets leading to exfoliation of the filler. Surfactants adsorb on the surface of the filler, and stabilize colloidal particles by the mechanism described by the DLVO (Derjaguin-Landau-Verwey-Overbeek) theory. The surfactant concentration for CNT dispersion generally needs to exceed a critical micelle concentration [34] and also needs to exceed the CNT concentration [34,35,36] in the solution.

Surfactants are most commonly used to disperse nanotubes in water. Common surfactants include sodium dodecyl benzenesulfonate (SDBS), sodium dodecyl sulfate (SDS), cetyltrimethylammonium bromide (CATB), sodium *n*-lauroylsarcosinate, nonyphenol ethoxylate. Other surfactants that have been successfully used to disperse nanotubes are cholate, oxycholate, and oligonucleotides. These are summarized in several reviews [37,38,39]. Attaching functional groups to the nanotubes such as hydroxyl [40] and carboxylic acids [41] improves dispersion in water. Other groups that enhance water dispersion include glucosamine [42], proteins [43], peptides [44], starch [45], amine-containing dendrimers [35], poly(ethylene glycol) [46], poly(styrene sulfonate) [47], and poly(vinyl alcohol) [48,49]. Certain organic solvents such as *N*-methyl pyrrolidone (NMP), α-caprolactone , and dimethyl formamide (DMF) have been shown to disperse carbon nanotubes [50]. Functionalization can also improve dispersion in solvents. Strong acids promote solubilization via protonation of the nanotubes [51]. However, during the functionalization process the use of an ultrasonicator, acids and strong oxidants can damage the properties of nanotubes such as shortening of the tube length or unzipping of the CNTs [52,53]. Below a critical length, nanotubes cannot transfer their load bearing ability (reduced aspect ratio) to the polymer matrix.

To increase interaction between the polymer and the filler, polymers can also be grafted to CNTs. There are two methods: the first, commonly referred to as “grafting to” approach, involves preformed polymer chains reacting with the surface of nanotubes. Examples include grafting of PS to oxidized single-walled CNT and multi-walled CNT, as well as PVA grafted by carbodiimide activated esterification reaction of oxidized nanotubes. Other techniques include reacting to oxidized nanotubes via esterification or amidation, nucleophilic addition, cycloaddition, condensation reactions, and sonochemical reactions. The main drawback of this first procedure is the low graft density caused by steric hindrance of the initially bound polymer chains on the nanotube surface to additional macromolecules. The second method, referred to as “grafting from” approach involves the polymerization of monomers on nanotube surfaces. In this process steric hindrance is not an issue and a high molecular weight polymer can be achieved. This process, however, requires accurate process control for the polymerization. “Grafting from” techniques include atom transfer radical polymerization (ATRP), reversible addition-fragmentation chain transfer, ring opening polymerization, free radical polymerization, cationic/anionic polymerization, condensation polymerization, reduction/oxidation polymerization, metallocene catalysis polymerization, electrochemical grafting, and nitroxide-mediated radical polymerization . These grafting techniques are summarized in several excellent reviews [54,55,56,57].

Depending on the exfoliation procedure, graphene can be dispersed with the aid of surfactant. Graphene can be dispersed in water with the aid of surfactants such as SDS, SDBS, CATB, and tetradecyl trimethyl ammonium bromide. Organic solvents such as DMF, NMP, and cyclohexanone have successfully exfoliated graphene [38]. Graphene exfoliation and dispersion in low boiling solvents such as chloroform, acetone, and isopropanol have also been reported [58]. Unlike in the case for CNT, the surfactant concentration does not have to exceed that of the graphene for dispersion. Surfactants such as sodium cholate perform better at concentrations below the critical micelle concentration [59] for dispersion. The dispersion quality of both CNT and graphene improves as the filler concentration decreases [36,60].

Graphene without functionalities interacts with polymer through van der Waals force, π-π stacking, and hydrophobic interactions [21]. These interactions are, however, weak. Functional groups can be inserted by oxidizing graphite using the Hummers method [61] using a mixture of sulfuric acid, sodium nitrate, and potassium permanganate. The resulting graphene oxide (GO), which generates homogeneous colloidal dispersions in water, alcohol, and organic solvents, possesses oxygen-containing polar functionalities such as carbonyl, hydroxyl, epoxides, and carboxyl groups [62,63]. The epoxide and hydroxyl groups are located in the basal plane of the graphene sheets while the carbonyl and carboxyl groups are located at the edges [64]. The oxygen groups in the graphene oxide reduce the van der Waals forces enabling water molecules to penetrate into the interlayer space of the platelets. Under the influence of the sonication and reduction in van der Waals force, exfoliation of GO occurs. The oxygen functionalities create electrostatic repulsion that prevents the re-aggregation of the exfoliated graphene oxide in water. GO has also been found to disperse in certain organic solvents such as *N,N*-dimethylformamide, *N*-methyl-2-pyrrolidine, tetrahydrofuran, and ethylene glycol [65]. Methods used in reacting polymers to nanotubes are also applicable in the case of graphene. Oxygen functionalities can form amide groups by reacting the amine group in *N*-ethyl-*N*′-(3 dimethyl aminopropyl) carbodiimide methiodide (EDC) with the carboxyl group in GO [62]. ATRP techniques have been used to graft poly (2-(dimethylamino)ethyl methylacrylate) to GO [66].

Graphene oxide is electrically insulating due to the disruption of the graphite structure during the oxidation process. This can be reversed by reducing GO by chemical, thermal, in alkaline environment, in supercritical water and by hydrogen plasma treatment [67]. However, the reduction of GO dispersion without stabilizer causes precipitation of graphite particles due to the rapid and irreversible aggregation of graphene sheets. Hence, prior to reduction, the surface of GO sheets needs to be modified by covalent modifications or non-covalent functionalizations [68]. Reducing agents that are generally used for the reduction of organic ketones, carboxylic acids, and epoxy functional groups can be used for the reduction of pure GO or functionalized GO [69]. Chemical methods include using reductants such as hydrazine [69,70], dimethyl hydrazine [70,71], hydroquinone [72], and sodium borohydride [73]. Thermal heating to above 2000 °C under various atmospheres (vacuum, Ar, H2, NH3) reduces graphite oxide as the oxygenated functional groups are removed during the release of gas molecules such as steam, carbon dioxide, and carbon monoxide [74,75]. Reduction of GO can also be achieved by electrochemical [76,77] or photocatalytic means [78]. The different techniques used to functionalize graphene are summarized in several review articles [67,79,80,81]. However, one drawback of reduced GO is that during the process lattice defects are incurred and this drops the modulus to approximately 220 GPa [82,83].

Non-covalent functionalization of graphene has been achieved via π-π stacking or van der Waals interactions. Such methods prevent any structural damage that affects the electronic properties. Graft and block copolymers have been used as compatibilizers for polymer/CNT composites. Here one chain of the copolymers interacts with the carbon nanofillers by π-π interactions while the other chain is miscible with the matrix polymer. This results in well dispersed CNTs in the polymer matrix. Graphene can be wrapped in poly(sodium 4-styrenesulfonate) that renders it soluble in water [71]. Aqueous dispersion of graphene was prepared using tetracyanoquinodimethane anion, pyrene derivatives, and sulfonated polyaniline. The tetracyanoquinodimethane stabilized graphene sheets were also found to disperse in DMF and DMSO [67,84].

The functional groups on graphene oxide open the possibility of interaction with polymer during processing. Hydroxyl terminated polymers can react with the carboxyl groups on the graphene oxide through esterification. GO can form nanocomposites with hydrophilic polymers such as poly(vinyl alcohol) (PVOH) [85], and poly(ethylene oxide) (PEO) [86]. The carboxyl and hydroxyl group of the GO reacts with organic isocyanates. These isocyanate modified graphene oxides have been known to form composites with polystyrene (PS), acrylonitrile-butadiene-styrene (ABS), and styrene-butadiene rubbers (SBR) [87]. Other polymers that have been incorporated with functionalized graphene include poly (methyl methacrylate) (PMMA), poly vinvyl acetate (PVA), PS, polyaniline (PANI), polyurethane (PU), nylon as well as polyesters such polycaprolactone (PCL), polyethylene terephthalate (PET) and polylactic acid (PLA) [21,84,87,88,89]. The effect of the degree of functionalization, the molecular structure, and the molecular weight of the functional groups on the mechanical properties of functionalized graphene were investigated. While the Young’s modulus was found to be insensitive to the molecular weight of the functional group, a decrease in the Young’s modulus with increasing levels of functionalization was observed [90].

Polymer chains can be grafted to the surface of modified GO—using the “grafting to” and “grafting from” techniques used to graft polymers to CNT surfaces. This technique increases interfacial interaction between the polymer and the GO through covalent bonding. Grafting of hydroxyl terminate polyvinylcarbazole to GO using diisocyanate [91], or PVA grafted to GO sheets by esterification in DMSO [85] are examples of “grafting to” techniques. “Grafting from” occurs during *in situ* polymerization. ATRP is an effective method used for grafting polymer chains [68,92].

For polymer clay nanocomposites, either the polymer or the clay needs to be modified. Natural clay is hydrophilic and can readily interact with polar polymers like PEO, and PEG. The interaction happens due to the formation of hydrogen bonds between the hydroxyl groups in PVA and PEG, or oxyethylene groups in PEO [93,94,95,96]. The extent of modification of these clay platelets affects the performance of the composite. Modifications to make MMT organophillic include surfactants such as alkyl and quaternary ammonium halides. Surfactants with a single long alkyl tail gave the highest level of exfoliation when combined with nylon, which decreased as longer alkyl chain surfactants are added [97]. Molecular simulations [98] indicate that the presence of in the alkyl chain of ammonium ions (-OH and -COOH) increased binding energies with nylon. A research group in the Toyota corporation used a solution of 12-aminolauric acid in concentrated hydrochloric acid to modify the surface of MMT with subsequent polymerization with ε-caprolactam to reinforce nylon [99]. However, nonpolar polyolefins have minimal attraction to polar silicate surfaces, and an increase in the number of alkyls on the surfactant improves dispersion [100].

**Figure 3:**
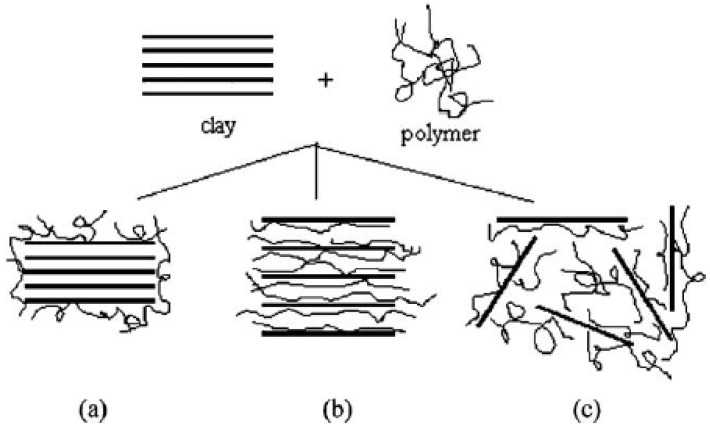
Schematic representation of different types of composite; (**a**) conventional composite; (**b**) intercalated composite; and (**c**) exfoliated composite. (Reproduced from reference [101], with permission).

Polymer clay composites can be classified into three types (Figure 3)—(i) immiscible or conventional composites; (ii) intercalated nanocomposites; and (iii) miscible or exfoliated nanocomposites. Complete exfoliation is seldom achieved in practice. Modification of MMT by quaternary ammonium compounds changes the morphology of conventional composite to intercalated [101]. In conventional composites, the clay platelets exist as aggregates or tactoids as in the original clay powder. The wide angle X-ray diffraction patterns for the composite are the same as that for the organo-clay powder. For a completely exfoliated clay, on the other hand, the X-ray peak is absent [22].

Clays are capable of modification through cation exchange, silane grafting, and adsorption of polar polymers [102]. Cation exchange depends on crystal size, the pH, and the type of exchangeable ions [103]. The amount of cations that can be exchanged depends on the amount of exchangeable sites and the structure of the silicate. The exchangeable cation affects the interaction between organic molecules and clay, particularly the likelihood of intercalation or exfoliation [104]. Organosilanes contains organic moiety where a covalent bond can be created between the silicate filler and the polymer matrix.

The ion-exchange reactions with cationic surfactants include primary, secondary, tertiary, and quaternary alkylammonium or alkylphosphonium cations. These surfactants can provide functional groups on the clay that react with the polymer matrix, or in some cases initiate the polymerization of monomers to improve the strength of the interface between the inorganic and the polymer matrix. The increased modulus and impact properties observed during the processing of nylon and clay are due to the formation of ionic bonds between the amine groups on nylon and clay sheet. These exchangeable cations affect interaction with organic molecules, which control the morphology (intercalation or exfoliation). Wilke [105,106,107] developed clays treated with trpylium or triphenyl hexadecylstibonium trifluromethylsulfonate or that containing oligomeric styrene, methyl methacrylate, or ε-caprolatone for reinforcing PS, PE, polypropylene (PP), PMMA or ABS as they are more stable than quaternary ammonium treated clay.

Unlike polyamides, more commonly used commodity polymers such as polyolefins do not have any polar groups capable of interacting with the aluminosilicate surface of the clay. One way to address the shortcoming is to add maleic anhydride grafted polyolefin to the mixture of clay and polyolefin. The maleated polyolefin fulfils the role of a compatibilizer between two immiscible polymers. This approach has been found useful for developing PP-clay [108,109] and PE-clay [100] systems. The use of maleic anhydride functionalized polymers gave better mechanical properties than when the compatibilizer was absent.

## 4. Nanocomposite Processing

The ultimate properties of the nanocomposite are dependent on the processing methods and processing conditions. Most composites can be processed using one or more of the following methods: (a) melt processing; (b) solvent processing; (c) *in situ* polymerization; (d) electrospinning; and (e) layer by layer (LBL) assembly.

Melt blending is one of the most economical and environmentally friendly methods of fabricating composites. This is the processing method of choice for most industries. The compounding is generally achieved in a single or twin-screw extruder where the polymer and the nanoparticle mixture are heated to form a melt. The mixer imparts shear and elongational stress to the process helping to break apart the filler agglomerates and dispersing them uniformly in the polymer matrix. Better dispersion is achieved with MWCNT than SWCNT [110]. The extruder is a versatile device whereby simply changing the screw configuration better control of shear and mixing is obtained. Higher shear rates tends to provide better dispersion. Production rates and material through-puts in a continuous extrusion process can be high. Another advantage of melt processing is that it does not require the use of organic solvents during processing. The compounded nanoparticle-polymer composite can be further processed using other polymer-processing techniques such as injection molding, profile extrusion, blow molding *etc*. Because of the large number of variables involved (temperature, screw-speed, residence time, and shear stress) the mixing process needs to be fine-tuned for optimal properties. Differing characteristics such as agglomerate structure, packing density, length to diameter ratio and purity affect the dispersibility of the nanotubes MNWT in polymeric matrix. In addition, the polymer matrix (specifically the melt viscosity) also affects the degree of dispersion. Unfortunately, the shear forces generated in most mixing equipment are not large enough to break and disperse the CNT in the polymer matrix efficiently. Special mixers where shear rates are an order of magnitude higher than obtained in a typical screw-extruder are often used leading to better dispersion and improved properties. However, the high shear also has the potential for degrading both the polymer and the CNT.

Most of the work reported in the literature has involved polymers such as low density PE (LDPE), HDPE, PP, PS, PMMA, polyamide, polyesters, and polycarbonate (PC). There are several reviews that detail the important findings [3,25,32,33,111,112,113]. Melt processing has shown modest improvement in mechanical properties. It has been reported [114] that intensity in the mixing section improves dispersibility over the kneading section. Polymers containing functional groups capable of reacting with functional groups on nanotubes improves dispersions [115]. Extensional flow gave better dispersion of nanotubes than shear flows [116].

There are limited studies on the melt blending of graphene and polymers. The low thermal stability of most chemically modified graphene and the low bulk density of graphene makes the use of melt processing difficult. Use of high shear melt mixing has been used to fabricate graphene based nanocomposite with PLA [117], PET [118], PP [119], nylon 6 [120], PC [121], PS [122], and elastomers [123]. However, high shear forces can cause buckling, rolling or shortening of graphene sheets [82], thus reducing its aspect ratio.

Melt mixing of clay with polymer has met with great success. There is a body of literature on melt compounding of clay and polymer. A wide range of polymers such as PS [124,125], polyolefins [126], polycarbonate [127,128], PCL [129,130], lactides [131,132,133], PMMA [107,134,135], polyamide [97,99,136,137,138], ABS [139,140], and PEO [141,142] have been successfully melt compounded with clay to obtain various degrees of exfoliation. The intercalation during melt processing of clay-PEO can be further improved by using microwave irradiation [143]. Liu *et al.* [144] obtained an exfoliated composite by compounding organic clay and nylon 6 using a twin-screw extruder. In general, the degree of exfoliation between nylon 6 and clay is quite high because of its excellent affinity for the silicate surface [22]. Stresses generated during melt blending can break up the clay aggregates. The greater the affinity between the clay and the polymer, the greater is the dispersion of the individual platelets in the polymer matrix. Polyolefins which have no polar groups to interact with clay often have to be modified. Polyolefins with maleic anhydride have better properties than unmodified polyolefins when compounded with clay platelets [100,108,145]. Longer residence times in the extruder leads to better dispersion [146]. Similarly, higher melt viscosity leads to higher stresses and better dispersion of the clay particles [22,146].

Solution mixing or solvent casting is another method of producing composites containing graphene or nanotubes and polymer. As the name suggests, solvent casting involves the agitation of the nanoparticle in a polymer that is dissolved in a solvent before casting in a mold and evaporating the solvent. Both thermoplastic and thermoset materials have been produced. Polymers such as PMMA [51], poly(vinyl alcohol) [49,147], polyhydroxyaminoether (PHAE) [148], PS [149], PE [150], PEO [151] and epoxy [152,153,154] with CNTs have been processed. The lower viscosity of the polymer in solution (as opposed to a melt) coupled with agitation by mechanical stirrer or ultrasonication aids in better dispersion of nanoparticles in the polymeric matrix. Different solvents, from aqueous to organic can be used. However, the removal of organic solvent after casting has environmental implication. Composites involving modified graphene and polymers such as PVA, PMMA, PP, PS, LLDPE, nylon, epoxy, PANI, and PU, have been prepared using the solution mixing and solvent casting method [84]. Solution casting involving clay often uses water soluble polymers such as PEO [96,155,156], lactide [157], and PVA [158,159] though casting using organic solvent [160] has also been attempted. The clay particles are swollen in the solvent. When the polymer solution is added to suspended clay during heating and dispersion, the polymer chains intercalate and displace the solvent within the interlayers of the clay stack. The polarity of the solvent is critical in facilitating the intercalation of the polymer into the space between the clay platelets [101].

A variety of CNT-polymer composite has been prepared using *in situ* polymerization. This technique can be used to produce both thermoset and thermoplastic materials. Here the nanotubes are dispersed in the monomer which is then polymerized. Dispersants may be added to assist in the de-agglomeration of the nanotubes [161]. Alternately functionalization [116,162] or polymer adsorption [163] techniques have been used to aid in dispersion. Polymerization is initiated by increasing the temperature, adding a chemical that initiates the reaction or by mixing two monomers. Since nanotubes are microwave absorbing causing an increase in temperature, microwaves have been used to induce polymerization [164]. One of the advantages of this technique is that it allows the grafting of polymer molecules on to the walls of the tube. The technique is useful in making CNT composites with polymers that are insoluble in most common solvents or are thermally unstable (thereby making melt processing difficult). Some of the composites developed include PE [165], PP [166], PMMA [167], PU [168,169], PCL [170], and PLA [171]. The *in situ* polymerization technique has been used to form composites of poly (vinyl acetate) [172], nylon 6 composite by bulk condensation polymerization of caprolactam in its mixture of GO [173], *in situ* synthesis of PU in DMF [123], emulsion polymerization of styrene in water with GO [174], or PMMA with GO [175].

The solvent casting method of making clay polymer composites has been attempted for water soluble polymers such as PEO, PVA, and poly(vinyl pyrolidone) (PVP), though solvents such as toluene [176], chloroform [156] and, acetonitrile [96,155] have been used. There have been problems with the quality of clay dispersion [158,177] and the volume fraction is fairly low and has not been extensively explored. *In situ* polymerization, on the other hand, is a versatile technique that has received increased attention. Heterophase polymerization in aqueous media is an attractive option [102], as it results in product with low viscosity compared to bulk polymerization and is environmentally friendly. Composite synthesis through emulsion polymerization involving PMMA [178], poly (styrene-acrylonitrite) [179], poly (styrene-co-butyl acrylate) [180], and polystyrene [181,182] have been reported. This route enables the synthesis of composite containing clay platelets located either at the surface or embedded inside the polymer particle. Compared to bulk polymerization and solution polymerization, emulsion polymerization leads to better dispersion of clay platelets in the polymer matrix after the removal of water [102].

**Figure 4:**
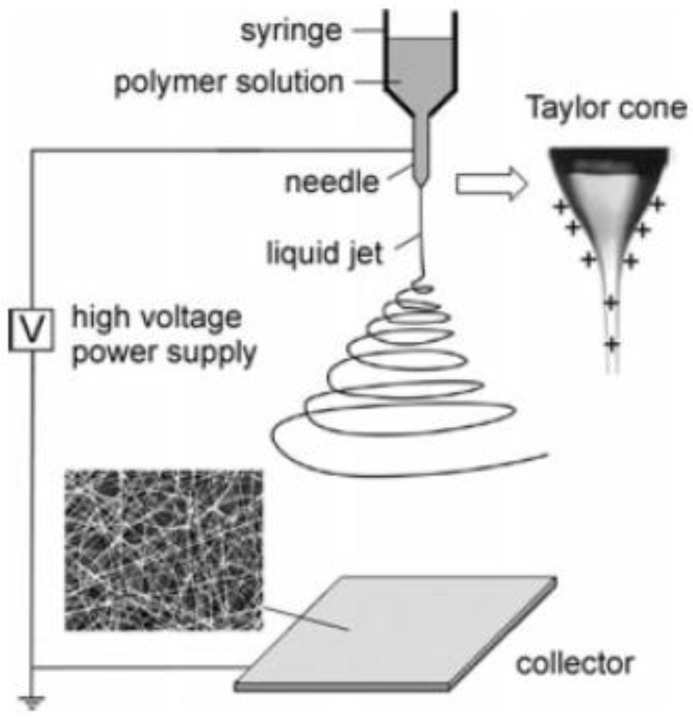
Schematic of basic electrospinning set-up with SEM image of nonwoven mat of poly(vinylpyyrolidone) nanofiber deposited on the collector (From Reference [187] with permission).

An alternate technique to fabricate polymer/nanofiller composite fibers is electrospinning. This technique allows the alignment of the CNTs along the fiber axis. The diameter of electrospun polymeric fibers ranges from tens of nanometers to several microns. The elements of a basic electrospinning unit include an electrode connected to a high voltage power supply that is inserted into a syringe-like container containing the polymeric solution. A schematic of the set-up is shown in Figure 4. Connected to the syringe is a capillary. The syringe-capillary set up can be mounted vertically [183], horizontally [184] or tilted at a defined angle [185]. A grounded collector plate, which is connected to the other end of the electrode, is placed at a distance of 10–30 cm from the tip of the capillary (Figure 4). The polymer solution at the end of the capillary upon the application of high voltage becomes charged. As the voltage is increased, a charge is induced on the surface of the liquid. Mutual charge repulsion leads to development of a force directly opposite to the surface tension. A jet is ejected from the suspended liquid meniscus at the end of the capillary when the applied electric field overcomes the surface tension of the liquid. Further increase in the electric field causes the hemispherical surface of the droplet at the tip of the capillary tube to elongate and form a conical shape known as the Taylor cone. When the repulsive electrostatic force overcomes the surface tension of the fluid, the charged jet is ejected from the tip of the Taylor cone. Within a few centimeters of travel from the tip, the discharged jet undergoes bending instability (Raleigh instability) and begins to whip and splits into bundles of smaller fibers. In addition to bending instability, the jet undergoes elongation (strain ~10^5^ and rate of strain ~10^3^ s^–1^) which causes it to become very long and thin (diameter in the range of nanometers to micrometers). The solvent evaporates, leading to the formation of a skin and solidification of the fluid jet followed by the collection of solid charged polymer fibers on the collector, usually in the form of non-woven fabric.

Parameters that affect the formation of nanofibers during the electrospinning process include (1) solution properties—viscosity, elasticity, conductivity, and surface tension; (2) system properties—hydrostatic pressure in the capillary, applied voltage, distance between tip and collecting screen, and (3) ambient parameters—solution temperature, humidity, and air velocity [186]. Comprehensive reviews on this topic can be found in several monologues [186,187]. Parameters that control fiber diameter are concentration of the spinning solution, electrical conductivity of the solution, and the feeding rate of the solution through the nozzle.

The potential of incorporating nanometer-sized particulates into fibers has made this process even more attractive for the production of composite fibers. The possibility of orientating the filler particles within the fibers during processing enables the possibility for manipulation of nanoparticles in which they are embedded and oriented. The critical material parameters for manufacturing such composite nanofibers include the geometry of the fillers, and the extent of homogeneous dispersion of filler within the polymer solution.

Graphene oxide has been electrospun with thermoplastic PU [188], polyimide [189], gelatin [190], PVA [191,192], nylon 6 [193], PANI [194], PVP [194], and PVA [195]. A small addition of GO increased both the strength and modulus of the composite [189,191]. Clay has been electrospun with PMMA [196], PVA [197], PEO [198], PVF [199], PLA [200], nylon 6 [201], and poly (ethylene-co-vinyl acetate) [202]. A number of CNT/polymer composites (mostly consisting of MWNT) have been successfully electrospun making it a versatile fiber processing technique. The alignment of the CNT in the polymer enhances the aspect ratio for reinforcing and increases the area for interfacial bonding [203]. Polymers that have been electrospun with CNT’s include polyacrylonitrile [204,205], PVA [206,207], PEO [206,208], PMMA [209], PU [210], PCL [211], PLA [184], PS [210,212], nylon 6,6 [213], and silk fibrion [214]. Significant increases in fiber modulus were reported upon the incorporation of nanotubes [184,205,210].

**Figure 5:**
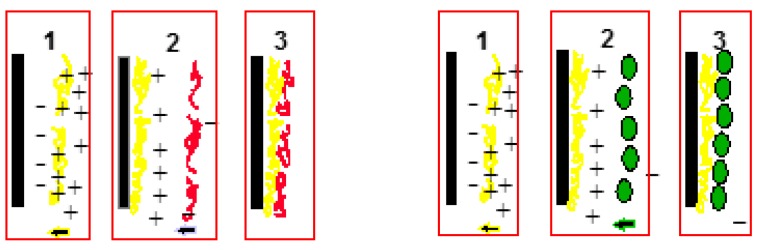
A negatively charged substrate is immersed in the solution of positively charged polyelectrolyte. The latter forms a submonolayer on the surface of the substrate, which switches the surface charge to positive. After rinsing with water, it is then immersed in the dispersion of negatively charged polyelectrolyte or nanoparticles. This results in the formation of a new layer, which switches the surface charge to negative. The whole cycle can be repeated as many times as is desired.

Another technique, often used to assemble multilayer and multi-material thin films is the layer by layer (LBL) assembly. The technique involves immersing a negatively (or positively) charged substrate in an oppositely charged polyelectrolyte (PE) which is adsorbed onto the substrate. After equilibrium is reached, the substrate is removed, rinsed, dried, and immersed in a negatively charged polyelectrolyte solution (Figure 5). This process is repeated until the desired thickness is achieved. The thickness of the film depends on the concentration of polymer in the solution, ionic strength, molecular weight of polymer, temperature during assembly, ionic strength, and pH [215]. The absorption of the polyelectrolyte is irreversible and charge overcompensation leads to charge reversal at the surface [216]. Different materials can be inserted between layers as long as they have the opposite charge.

One of the advantage of LBL assembly is the high level of dispersion of nanoparticle into a composite which occurs as a result of direct absorption of nanoparticle from a solution to a solid state without phase segregation. The first report of LBL assembly of clay nanosheets with polyelectrolytes was in 1994 [217]. Graphene-based LBL assembly was reported in 1996 using non-exfoliated graphite oxide platelets and PE [218]. GO sheets have been incorporated into LBL assembly through stable GO suspensions containing negatively charged GO-COO^−^ or positively charged GO-NH_3_^+^ prepared by introducing amine groups on the surface of negatively charged GO sheets [219].

Initial results from multilayered film assembly showed linear growth of mass and film thickness for [220]. In these films each polyelectrolyte interpenetrates only its neighboring layers. However, films that experience exponential growth have also been reported [221]. This exponential growth pattern was attributed to the vertical diffusion of polyelectrolyte into the film. Diffusion is controlled by the molecular weight (MW) of the polyelectrolyte, with higher MW diffusing much more slowly. Other factors that affect diffusion include polymer charge density, and the nature of chemical groups present on the polymer.

LBL assembly initially focused on construction films based on electrostatic interaction, subsequent works have focused on developing LBL composites based on hydrogen bonding [222,223], charge-transfer interactions [224,225], coordination bonding and covalent bonding [226,227,228]. Through hydrogen bonding, a number of additional materials can be incorporated into multilayered composites in a water solution or organic phase. A number of polymers can act as donors and acceptors for hydrogen bonding. Hydrogen-bonding multilayer film assembly is based on the alternate deposition of polymers containing a hydrogen bond acceptor and a hydrogen bond donor, respectively. Compared to films assembled using electrostatic attractions, the pH range where hydrogen bonded multilayers form stable films are limited. Disassembly can be achieved through fine tuning the pH by varying the hydrogen-bonding pairs or the conditions under which the layers are assembled. However, the films can also be made stable by cross-linking using chemical, thermal, and photochemical techniques.

Covalently bonded multilayered films have also been assembled using the LBL techniques. The presence of covalent bonds imparts stability to the films. The strength of the composites depends on the strong adhesion between the two polymers. The films can be assembled in organic solvents, where electrostatic interaction is impossible because a polycation and polyanion will form a salt and precipitate in solution. LBL composites containing carbon nanotubes have been assembled using electrostatic interactions [229,230] or hydrogen bonding [231,232]. Covalent cross-linking can increase the modulus [233,234] and stability of the films. This is achieved by using polymers that have functional groups that are capable of reacting with one another or can react with a bi-functional agent (using diamines, diimides or dialdehydes).

The general concept of LBL assembly makes this a versatile method for combining different kinds of materials (CNTs, clays, nanoparticles (NPs), polymers, proteins, *etc.*) in making nanocomposites. This technique has the capability of controlling the morphology at nan oscale level, to enable a more compact and ordered structures compared to other solution-based methods. LBL assembled thin films can find applications in transparent conducting films, field effect transistors, and supercapacitors. Slow deposition speeds and cumbersome assembly process remain the main drawback of this process.

## 5. Properties of Nanocomposites

1. Reinforcement: While it is well know that nanofillers increase the mechanical properties of composites, it is widely recognized that the excellent properties of nanofillers have yet to be realized. This is particularly true at higher volume fraction. Using the Halpin-Tsai [235] equation, for an aspect ratio of 1000 for 1D nanotubes or 2D platelets, a modulus enhancement by a factor of six is predicted at 0.01 filler volume fraction. Molecular simulations predict similar reinforcing potentials for aspect ratio of approximately 100 [236]. Relative improvements in modulus are expected to be much higher than the increase in tensile strength. Strain at break generally decreases with nanofiller loading. For nanotubes, properties generally increase with increasing nanotube content at low volume fractions but then decrease at higher fractions due to issues related to dispersions and agglomeration. It has also been reported that higher surface area leads to better reinforcement [237], except for single walled nanotubes which the authors attributed to poor dispersion. Even with improved adhesion and dispersion in the polymer matrix, the nanotubes remain randomly dispersed. Attempts have been made to align nanotubes to increase reinforcement. Alignment techniques include melt drawing [9], polymer stretching [148,238,239], alternating-current electric field [240,241,242,243], surface acoustic waves [244], direct-current electric field [241,242,243,245] and magnetic fields [246,247,248]. Studies [9,238,239,249,250,251] have shown that in composites where the nanotubes were aligned, a significant increase in the modulus was obtained over non-aligned composites. However, alignment of nanotubes in the composite also caused anisotropy—with improvement in the perpendicular direction being significantly less [252]. The use of magnetic field as a technique to align nanotubes gave conflicting results on modulus enhancement [246]. There is a great body of literature that covers various aspects of mechanical property enhancements of different polymer systems for various types of nanotubes. These have been summarized in several excellent reviews [54,111] and monographs [25]. The mechanical properties of selected nanotube polymer compositions are shown in Table 1. It is difficult to compare the results as the type of nanotube, the method of processing, the type of functional groups, the aspect ratio, and the type of polymer all affect the properties.
  As in the case of nanotubes, modulus of graphene filled nanocomposites increased with loading fraction (Table 2). Results indicate that the strength of the interface is critical to the enhancement of the mechanical properties. Modulus increase is more pronounced for elastomeric matrices due to their lower intrinsic modulus. Studies have shown that the mechanical reinforcement of graphene is superior over fillers such as carbon black or single wall nanotube [88]. For epoxy polymers, functionalized graphene sheets have better fracture toughness, fracture energy stiffness, strength, and fatigue resistance at lower loading fractions as compared to nanotubes. [24,253]. Tensile strength has generally also been shown to increase with increase in graphene content, though there are instances when tensile strength decreased [254]. Elongation generally decreased or remained the same. As with nanotube based composites, the improvement in mechanical properties observed falls well short of that predicted theoretically.
  The mechanical properties of nanoclay mirrortrends are seen in nanotube polymer composites or graphene polymer. Addition increases modulus and tensile strength but decreases elongation at break. However, with clay, the relative reinforcement [2] for a given volume percent of filler in the composite is significantly lower than for graphene composite [88] or carbon nanotube based composite [54]. The tensile modulus of a polymeric material has been shown to be significantly improved when nanocomposites are formed with either pristine or organically modified clays. For nylon 6, tensile strength increased by 42% and modulus by 90% [255,256]. Interestingly, the stiffness increases with the increasing molecular weight of the matrix at any given loading, even though all the moduli of the neat nylon 6 are quite similar [257]. Similarly, the increase in strength relative to the neat matrix for the high molecular weight composite is nearly double compared to that of the low molecular weight composite. While nylon 6 interacts with silicate surface through hydrogen bonding, nancomposites with polyolefins require modification of both polymer and clay. For PP-clay composition, the modulus increases with clay content untill 3 wt% of clay, after which with an increase in clay content the modulus shows minimal improvement [109]. When MA functional groups are incorporated in the PP the stresses are much more effectively transferred from the polymer matrix to the inorganic filler, and thus a higher increase in Young’s modulus was observed. For maleic anhydride functionalized LLDPE, modulus increase is higher with MMT with two alkyl tails [100]. The addition of LLDPE-g-MA is advantageous with clay content of greater than 2.5 wt%.
  Mechanical properties of thin films developed using LBL have shown promise in terms of mechanical reinforcement. Mamedov [233] used poly(ethyleneamine) (PEI) as the polycation and acid modified SWNT or poly(acrylic acid) (PAA) as the polyanion. The film was heated and cross-linked using glutaraldehyde. The tensile strength of a 40-layer PEI/SWNT/PAA film was reported to be 220 ± 40 MPa while that of PEI/PAA (without CNT) film of the same number of layers was 9 MPa. The modulus of the composite containing SWNT was 35 GPa. These values are better than any known engineering plastics or are obtained by blending with carbon fillers and are similar to those of ceramics. Hu [258] reported linear increase in modulus of silk fibrion graphene thin film with increasing graphene oxide. Kulkarni *et al.* [259] observed 500% increase in break energy, eight fold increase in modulus, 120% increase in modulus, and increase in ultimate strain in LBL of negatively charged graphene oxide in a polyelectrolyte multilayer. The measured modulus exceeds that predicted by mathematical models. PDDA/MTM multilayers have high strength, flexibility, and resistance to crack propagation [260]. Free standing films of 50, 100, 200 bilayers of PDDA/MMT displayed tensile strength of ~100 MPa and *E* ~ 11 GPa—the increase was 10× and 50× over virgin polymer [261]. PVA/MMT films covalently cross-linked using glutaraldehyde [234], displayed tensile strength of 400 MPa and *E* = 106 GPa.
2. Electrical Conductivity: The excellent electrical conductivity of CNT’s and graphene can be exploited to make traditionally insulating polymer matrices into electrically conductive materials for various applications in conductive adhesives, antistatic coatings, and films. There is a critical loading (percolation threshold) when the composite transitions from an insulator to a conductor due to the formation of a continuous conducting network. Below the percolating threshold, the electrical properties are dominated by the dielectric matrix and hence the composite is non-conductive as the fillers do not form a continuous network for electrons to flow. Beyond the percolation threshold, a small increase in loading will result in a significant increase in conductivity. As filler content increases, the fillers begin to form a contact with each other. At percolation threshold, conduction paths are created in the insulating matrix causing an increase in conduction. However, beyond a certain level of filler concentration a plateau in conductivity is reached. Factors affecting percolation threshold include aspect ratio, functionalization, processing, polymer type, dispersion *etc.* [53]. For example, it has been reported [292] that nanocomposites prepared by *in situ* polymerization showed significant increase in electrical conductivity compared to melt blending. There are no clear trends regarding the type of polymer and its effect on conductivity, although electrically conducting polymers have higher conductivities in the case of CNTs. According to theoretical prediction [293], rod-like structures percolate at one half the volume fraction of disk-like structures. However, there are examples that indicate that graphene has a lower electrical percolation threshold than CNTs [294]. Contradicting results have been published concerning the effect of aspect ratio on the percolation threshold [295,296]. Percolation threshold becomes greater as particles are aligned parallel. In general, SWNT composites have lower conductivities than MWNT composites due to the high contact resistance in SWNT composites because of smaller diameter. Using a similar processing method it was observed that the conductivity of graphene PS films had conductivity several orders of magnitude lower than films with CNTs [297]. For aligned CNT/epoxy composites, the electrical percolation threshold was 0.0025 wt% [298,299]. In comparison, the lowest percolation threshold for graphene based composite was 0.19 wt% for PS solvent blended with isocyanate-treated GO [69,87]. Electric conductivity of CNT and graphene based composites are summarized in several review [54,84,88,300].
3. Thermal Conductivity—The thermal conductivity of carbon nanotubes has been estimated to be in the range between 650 and 10,000 W/mK. The thermal conductivity of a typical polymer ranges between 0.3 and 0.4 W/mK. It was anticipated that nanotube based composites would experience a significant increase in thermal conductivity (similar to electrical conductivity enhancements). In reality the increase has been rather modest (typically less than 1 W/mK). The reason can be attributed to large resistance to heat transfer at the nanotube-polymer interface. Functionalized nanotubes gave higher thermal conductivity than unfunctionalized tubes indicating that higher dispersion aids conductivity [301]. There have been few studies relating to the thermal conductivity of graphene/polymer composites. The most improvement in the thermal conductivity of graphene based nanocomposites were those where the nanocomposites were produced via *in situ* polymerization using chemically modified graphene [92,302]. Since thermal conductivity of composites increases linearly with filler content, a 20 fold increase was obtained by loading the composite with nanofiller [303].
4. Thermal Stability: Polymers have a high thermal expansion coefficient when compared to metals. The addition of fillers like clay or nanotubes of graphene reduces the thermal expansion of polymers by constraining the movement of a significant volume of polymer chains because of their interaction with the filler. Graphite has a positive thermal expansion coefficient and when incorporated into polymers does not reduce the expansion of polymers [6]. However, incorporating reduced GO or SWNTs into resins displayed the effect of reduced thermal expansion [304]. Graphene oxide or single walled carbon nanotube have a negative thermal expansion coefficient and hence, composites containing GO or SWNT increase the thermal stability by decreasing the coefficient of expansion. Liu *et al.* [305] reported that the degradation temperature of PS increased from 400 to 450 °C when impregnated with graphene sheets. Thermal stabilities of graphene/PMMA were reported to be higher than that of PMMA [285]. Clay nanocomposite samples prepared using injection molding displayed anisotropy; the expansion coefficient in the flow direction was lower than in the perpendicular direction [127,306] and this difference was attributed to the orientation of platelets in the respective direction. Similar results were observed when MMT was aligned using magnetic or electric field [307,308].
5. Glass Transition Temperature: Fillers can be an impediment to the motion of polymer chains due to the interfacial interaction between the polymer and the filler. Studies have confirmed that both *T_g_* and the breadth of the transition can be affected by nanofillers [309,310]. Factors that influence *T_g_* include sample thickness [311,312], sample preparation and measurement [313], nanoparticle dimension [309,310,314], and chemical structure of the polymers [315]. The interaction of the filler with the surface will determine the degree of change of T_g_. Surfaces that interact strongly with the polymer causes an increase in *T_g_* [316,317] relative to the bulk. The unadsorbed material can have the same or lower *T_g_* than the bulk depending on the nature of the adsorbed layer. Even hydrogen bonds at the polymer-substrate interface can increase *T_g_* relative to bulk values [313,317]. Graphene platelets with higher aspect ratio, higher surface roughness, and which are well dispersed in the polymer lead to a composite with higher *T_g_*. Liao [313] observed that solvent and melt blending processes lead to insignificant changes in the *T_g_* of polymer-graphene or polymer-GO composites, while *in situ* polymerization with unmodified graphene or solvent blending with chemically modified graphene or GO causes an increase in *T_g_*. The authors attributed this to the covalent bonding between the graphene and the polymer. The type of polymer also affects how and whether the *T_g_* increases, decreases or remains unchanged. For example, nanospherical silica showed an increase in *T_g_* with PVP, decrease in *T_g_* with PMMA, while *T_g_* was unchanged with PS [318]. Mixing a polymer and nanotube can increase or decrease *T_g_* depending on the surface functionalization of nanotubes. However, polyimide mixed using a dispersion-reaction scheme with non-functionalized depressed *T_g_*, with acid-functionalized, and amine-functionalized tubes, elevated *T_g_* [319]. Another important effect due to nanotube polymer interaction is the amount of material participating in the glass transition [320]. Also, nanotube dimension does not affect polymer/nanotube interaction. However, nanotubes can also affect the growth rate of crystals as they alter chain mobility and provide impediment to growth [110]. Higher *T_g_* in some exfoliated and intercalated polymer clay nanocomposites has been attributed to the large interlayer distance between the clay platelets and the strong polymer—filler interactions that exist in the system [321,322,323]. When the interlayer distance is less than the characteristic length of polymer chains for relaxation, *T_g_* is either depressed or absent [125,324].
6. Barrier and Membrane Separation Properties—Composites containing fillers with large aspect ratio can impede and alter the diffusion path of penetrating molecules. Well dispersed fillers create a tortuous path for permeants to travel. A decrease in gas permeability, that is independent of the type of gas [99] was observed for clay reinforced composites. A 1%-loading of clay in PET showed a two fold reduction in O_2_ permeability. Messersmith and Giannelis [325] reported that the water vapor permeability of PCL clay nanocomposites decreased over neat PCL. Permeability was also observed to decrease as the aspect ratio of the platelets increased. Defect-free graphene sheets are impermeable to gas molecules [15], and hence, graphene polymer composites films can be used as protective elements in electronics and fuel cells that are sensitive to the presence of gases such as oxygen and moisture [123,326]. GO conjugated polymer nanocomposite films have been shown to significantly reduce oxygen and carbon dioxide permeation [326,327]. Graphene polyimide composite films have been reported to display high moisture barrier properties [328]. It has been reported that modified GO reduced the permeability of thermoplastic polyurethane more than modified MMT platelet layers at similar loadings.
7. Flammability Resistance: Polymers will burn easily compared to metals or ceramics. Above a certain temperature a polymer decomposes releasing gaseous products that react with the oxygen in the air and burn. Well dispersed nanoplatelet/nanotube in the polymeric matrix capable of forming a continuous network, form a protective layer on the surface. This protective layer which acts as a heat shield, in turn, prevents the gaseous degradation products from diffusing through it and reacting with the oxygen in the air. Nanofiller impregnated polymer shows a significant reduction in the maximum heat release [329] compared to neat polymer, though the total heat release remained unchanged. Aspect ratio of dispersed silicate layers and the processing method have a strong effect on the fire-retardant properties [330,331]. Both clays and nanotubes have been investigated as flame retardants. Nanotubes have been reported to be more effective retardants [29,332] over clay. However, because of the heat localization due to the high thermal conductivity and low specific heat, time to ignition is lower with nanotube addition. Poorly dispersed nanofillers or low concentration of fillers result in the formation of a discontinuous network leading to much poorer flame resistance [29].

## 6. Future Outlook

Carbon based nanofillers are relatively at an early stage of development. Nanoclay based nanocomposites have met with success. However, there are several fundamental challenges that need to be addressed before complete understanding of the nanofiller in polymer composites is attained.

1. It has been well documented that dispersion of nanofillers is critical in achieving properties of nanocomposites. However, many of the processing techniques used to manufacture these composites are not economically viable. Solvent processing, LBL assembly, and electrospinning while resulting in better dispersions of nanofiller in the polymer are not cost-effective. Melt processing, the only economically viable processing technique, generally leads to poor dispersion and less than optimal properties.
2. The problems associated with mechanical reinforcement of melt processed composites need urgent attention. At higher volume fractions, SWNT remains agglomerated. MWNTs are easy to disperse at much higher loading. There is an increase in interfacial area with decrease in tube diameter. For example, at 0.5 wt% of nanotubes MWNT has 70% of the interfacial area of SWNT [29]. Hence, SWNT lose their intrinsic advantage of higher aspect ratio. Similarly, composites at higher loading do not perform as well as at lower loadings. This puts a ceiling on the magnitude of reinforcement.
3. Development and quality of polymer composites with CNT, graphene or clay depend upon a number of factors such as types of CNTs (MWCNT or SWCNT), layers of graphene or clay, purity, length of CNTs, diameter and length of CNT (aspect ratio), loading of nanofillers, dispersion in the matrix, alignment (tough to align graphene or nanoclay), and interaction between the polymer and the nanofiller. However, there are no systematic studies that compare the effect of aspect ratio, nanofiller purity, degree of functionalization, and type of functional group on the properties of the composite. For example, minimal reinforcement is obtained from graphene flakes with an aspect ratio of 1000, while both modulus and strength doubled for graphene with an aspect ratio of 2000 [333].
4. Most work has been conducted using a single type of nanofiller in a polymer matrix. Simultaneous incorporation of different nanofillers may significantly enhance the properties of composites. The synergy in properties between multiple nanofillers needs to be investigated. For example, 1D fillers might interfere with the stacking of 2D platelets. Incorporating CNTs into glass fiber composites inhibits crack formations due to the large density of nucleation sites provided by CNT [334].
5. Load transfer between nanofiller and polymer has been achieved by both non covalent and covalent modifications with functional groups. Introduction of covalently functional bonds disrupts the π conjugation of CNTs and graphene, leading to a negative effect on the electrical properties of the resulting composites. It has been reported that a combination of non-covalent and covalently fuctionalization on CNT can enhance compatibilizer-polymer interaction leading to better mechanical and electrical properties [335,336]. Hence, it is critical to develop an understanding of the interface between the non-covalently functionalized CNT/graphene and the polymeric matrix to enable simultaneous enhancement of both mechanical and electrical properties of the composites.
6. It has been reported that nanofiller can act as nucleating agent and affect polymer crystallinity [113,337,338]. There should be more attempts to correlate the extent of change crystallinity with the mechanical properties of composite. Similarly, the effect of surfactants on *T_g_* of nanoparticles should be careful investigated [313].
7. Modification of organoclays that are stable at higher temperatures are also desirable. Organically modified clay starts to decompose at approximately 180 °C [339,340] This would enable the use of high temperature polymers like PEEK.

## 7. Summary and Conclusions

The current progress on the state of nanoparticle filled polymer nanocomposites and their potential application has been reviewed. The development of nanofiller reinforced polymer matrix has been an active area of research for well over two decades. Amongst the various nanofillers used for reinforcing, clay (montmorillonite MMT), carbon nanotube and graphene have been the most studied. The research field initially started with polymer-clay nanocomposites in the late 80s, followed by polymer-carbon nanotube nanocomposites in the late 90s. Graphene based composite research papers started around 2006 and have experienced exponential growth since 2010.

The reinforcement of polymeric systems by nanoscale sized fillers has opened up the possibility of improving modulus and strength of composites using a much lower filler content. The extent of reinforcement depends on the filler type, the type of functional group on the filler, the aspect ratio of the filler, the amount of filler, the type of polymer, and the method of processing. Homogeneous dispersion of the nanofiller in the polymeric matrix and strong interaction between the filler and the polymer is absolutely necessary for good reinforcement. At higher nanofiller content, composite properties decrease indicating difficulties in dispersing the fillers.

Alignment of nanofiller in the matrix increases mechanical properties but causes anisotropy. The alignment is much easier in the case of nanotubes because of their 1D structure. Composites films made with alternate layers of nanofillers and polyelectrolyte have shown great promise in terms of mechanical properties. These fillers can have high filler loading. The LBL assembly technique also minimizes damage to the nanofiller during assembly. Solution based techniques provide better mechanical reinforcements than melt based techniques. Large scale use of polymer/nanofillers can only be possible if the nanofillers are easily dispersed in the melt using conventional polymer processing such as extrusion and injection molding or fiber spinning in both melt and solution phases.

Covalently functionalized nanotubes provide better polymer reinforcement, allowing for better stress transfer between the polymer matrix and the nanofiller. However, covalent functionalization can destroy intrinsic electrical properties of CNT [111], requiring higher percolation threshold. CNTs can improve the thermal conductivity, flame retardancy, and the thermal stability of the composites.

Graphene based composite is attractive as it leads to improved mechanical properties, electrical conductivity, thermal conductivity, and thermal stability over neat polymers (similar to CNTs). Graphene filled polymers showed as much as 50 °C or larger increase in thermal stability. The percolation threshold of graphene was comparable to that of carbon nanotubes [87]. The conductivity depends on the method of synthesis and on the surface modification, particularly on the number of defects (reactive sites) generated during the oxidation-reduction process on the surface [88]. Homogenously dispersed graphene also reduced barrier properties. One barrier to increased use is the low bulk density of graphene sheets that make handling difficult.

Like the other nanofillers, the mechanical and barrier properties of nanoclay-polymer nanocomposites depend on the extent of dispersion of the silicate layers in the polymer matrix. Nanoclay can be modified by a wide range of organo-modifiers to enhance compatibilization with different polymers and help achieving the intercalation and exfoliation morphologies necessary for achieving the best mechanical properties. Processing can be performed using three primary techniques: solution blending, *in situ* polymerization, and melt blending or extrusion. Melt intercalation is considered the most promising approach for the fabrication because of its versatility, compatibility with current polymer processing equipment, and environmental friendliness due to the absence of solvents. Addition of pristine and organically modified clay increases the thermal stability of polymers. One drawback of clay concerns its stability at high temperatures.

LBL assembly offers promise as it offers the ability to control the morphology at the nanometer scale and allow a much higher loading of nanofillers compared to other processing techniques. Such morphological control has enabled composites to attain record properties. Furthermore, LBL technique can be used to incorporate inorganic nanoparticles, and nanotubes into organic polymers. Processing techniques such as elctrospinning or LBL assembly are unlike to find widespread usage in industrial settings due to their low production rate.

While clay based nanocomposites have found application in the automotive industry, there has been an unfulfilled expectation in the area of CNT/polymer nanocomposites, primarily due to high material costs and difficulties in processing. Cost remains an issue with both nanotubes and graphene. Pricing for research grade nanotubes range from around $5/g for MWCNT to $75/g for SWCNT [341,342]. Graphene oxide costs $100/g [341,342]. Nanoclay is relatively less expensive and melt-compounded composites containing nanoclay have found usage in industrial applications such as in automobiles [22]. Carbon nanotube and graphene based composites might find some applications in advanced composites, replacing carbon fiber, or even in the electronics area.

## Figures and Tables

**Table 1:** Mechanical properties of selected carbon nanotube (CNT) based polymers.

Polymer	CNT Type	wt(%)	Processing Method	σ/σ_0_	*E*/*E*_0_	Reference
PS	MWCNT	1.00	Solution Casting	1.25	1.42	[31]
PS	MWCNT	5.00	Solution Casting	1.5	2.2	[149]
PS	MWCNT	40.00	Melt Mixing	1.1	2.0	[262]
LDPE	MWCNT	10.00	Melt Mixing	1.56	1.89	[263]
LDPE	MWCNT	3.00	Ball Milling	3.50	1.3	[264]
PP	SWCNT	1.00	Solution mixing-fiber spinning	1.5	2.0	[265]
PP	SWCNT	0.75	Shear Mixing	1.15	1.4	[266]
PP	MWCNT	0.25	Melt fiber spinning	1.1	2.3	[267]
PP	MWCNT	1.00	Melt fiber spinning	5.0	3.7	[268]
PMMA	Oxidized MWCNT	5.00	*In situ* bulk polymerization	1.30	–	[269]
PMMA	Oxidized MWCNT	1.50	*In situ* bulk polymerization	1.75	–	[270]
PMMA	MWCNT	1.00	Melt Extrusion	1.0	1.0	[271]
PMMA	SWCNT	2.00	Solution casting	1.9	–	[51]
PMMA	PMMA-*g*-MWCNT	0.15	Solution casting	3.6	1.9	[272]
PVA	Hydroxy-modified SWCNT	0.80	Solution casting	1.47	1.79	[49]
PVA	Oxidized MWCNT	0.20	Solution casting	1.52	1.46	[273]
PVA	Oxidized SWCNT	0.20	Solution casting	1.42	1.29	[273]
SBR	MWCNT	10.00	Solution casting	4.0	5.0	[274]
Nylon 6	MWCNT	1.00	Melt Blending	1.2	1.1	[275]
Nylon 6	MWCNT	2.00	Melt Blending	1.62	2.14	[276]
Nylon 6	Oxidized SWCNT	0.5	*In situ* polymerization	2.5	3.5	[277]
PI	Oxidized MWCNT	0.38	Solution casting	1.6	1.5	[278]
PI	Oxidized MWCNT	1.00	Solution casting	1.23	1.40	[279]
PI	SWCNT	1.00	Melt Extrusion	1.0	1.5	[280]
PU	Oxidized MWCNT	1.50	*In situ* condensation	1.3	2.4	[169]
PU	Oxidized MWCNT	2.00	*In situ* condensation	1.15	1.4	[281]
PU	Oxidized MWCNT	4.00	*In situ* condensation	1.4	2.3	[282]
Epoxy	SWCNT	5.00	Solution casting	1.07	1.0	[283]
Epoxy	MWCNT	0.5	Solution casting	1.62	1.54	[154]

Notes: MWCNT—Purified MWCNT, SWCNT—Purified SWCNT; σ_0_—tensile strength of pure polymer; *E*_0_—modulus of pure polymer.

**Table 2:** Mechanical properties of graphene.

Polymer Matrix	Graphene Type	wt(%)	Processing Method	σ/σ_0_	*E*/*E*_0_	Reference
Epoxy	Expanded	1	Sonication	0.8	1.08	[254]
	Expanded	1	Shear	0.93	1.11	
	Expanded	1	Sonication and Shear	0.94	1.15	
PMMA	Expanded	21	Solution casting	–	1.21	[284]
	GNP	5	Solution casting	–	2.33	[285]
	GO	–	*In situ* Polymerization	–	1.54	[286]
	TRG	1.2	Solution mixing	1.80	–	[285]
HDPE	Expanded	3	Melt processing	1.04	2.0	[287]
PVA	GO	0.7	Solution casting	1.76	–	[288]
	Graphene	4.5	Solution casting	2.5	–	[289]
	GO	6.3	Solution casting	1.7	2.23	[290]
TPU	Graphene	13.3	Solution casting	–	3.0	[291]
	TRG	4.0	Melt	–	3.5	[123]
		4.0	Solution casting	–	7.8	
		3.78	*In situ* polymerization	–	3.1	
PC	TGO	2.5	Melt Processing	–	1.21	[121]
PC	Graphite	15	Melt Processing	–	2.48	

Notes: TRG—Thermally Reduced Graphene; GNP—Graphite Nanoplatelets; TGO—Thermally Exfoliated Graphene Oxide. σ/σ_0_ is the normalized tensile strength (tensile strength of composite/tensile strength of polymer matrix); *E*/*E*_0_ is the normalized modulus (modulus of composite/modulus of polymer).
